# Surgical treatment of developmental dysplasia of the hip in children – A monocentric study about 414 hips

**DOI:** 10.1051/sicotj/2022030

**Published:** 2022-06-29

**Authors:** Mohammed Tazi Charki, Hicham Abdellaoui, Karima Atarraf, Moulay Abderahman Afifi

**Affiliations:** 1 Department of Pediatric Orthopedic and Traumatology, University Hospital Hassan II – Sidi Mohamed Ben Abdellah University – The Faculty of Medicine and Pharmacy of Fez Boite Postale 1893 – KM 2.200 Route Sidi Harazem Fès 30070 Morocco

**Keywords:** Developmental dysplasia of the hip, Children, Open reduction, Outcomes

## Abstract

*Introduction*: No consensus exists about the open reduction of developmental dysplasia of the hip (DDH; age of surgery and the need for additional bone surgery). We report clinical and radiological outcomes of a large monocentric study. The objectives are to analyze outcomes and to give recommendations. *Materials and methods*: This was a retrospective review of 414 hips (301 patients) operated on for DDH between 2010 and 2018. The mean age at the time of surgery was 34.6 months (14–96 months). In all, 72 hips had open reduction (OR) alone, 130 had OR with femoral osteotomy, 37 had OR with pelvic osteotomy, and 175 hips OR was associated with femoral and pelvic osteotomy. The mean follow-up was 6.5 years (3–10 years). Clinical outcomes were evaluated according to Mckay’s classification. The acetabular index was measured, and Severin classification was used for radiological outcomes. Reduction failure and residual dysplasia were noted, and avascular necrosis of femoral head (AVN) was assessed according to Kalamchi and MacEwen classification. *Results*: At the last follow-up, 331 hips (80.2%) had good clinical results, and 319 (77%) had satisfactory radiological results. The AI measured on the last follow-up radiograph was ≤25° in 350 hips. AVN was noted in 83 hips (20%). Redislocation was founded in 53 hips (12%). Overall: 293 hips (72%) had stable reduction without AVN with good clinical and radiological outcomes. *Discussion*: Clinical outcomes are better and the risk of AVN decreases significantly when a femoral osteotomy is performed. There were better radiological results when pelvic osteotomy was performed. The rate of residual dysplasia was higher when pelvic osteotomy was not performed. We recommend a femoral shortening osteotomy for high dislocations (Tönnis 3 or 4) for children over 18 months and a pelvic osteotomy for children over 36 months or over 18 months with an acetabular index > 25°.

## Introduction

Developmental dysplasia of the hip (DDH) is one of the most frequent deformities in children [1]. The treatment is initially conservative based on orthopedic devices, but as children grow up, these methods are ineffective due to soft tissue contractures and bone deformities. Consequently, surgery should be considered [2, 3]. In our context, despite screening efforts, DDH is often diagnosed late at walking age.

The aim of surgery is to achieve a stable and concentric reduction while limiting the risk of complications, especially avascular necrosis of femoral head (AVN), redislocation or recurrence of acetabular dysplasia, and the need for secondary procedures [4, 5]. Surgery involves an open reduction of the femoral head with resection of the redundant capsule and the release of intra- and extra-articular soft tissues. Pelvic and/or femoral osteotomies may be necessary to maintain a stable and concentric reduction and avoid complications [6]. However, due to a lack of international consensus and guidelines regarding age at surgery and the need for additional bone time (femoral and/or pelvic), choice between these procedures remains controversial and is determined by surgeons and departments experience.

We conducted a monocentric retrospective study of 414 hips operated on for DDH over a period of 8 years with 6.5 years of follow-up. This study aims to present outcomes of a large series with clinical and radiological analysis, identify the different factors influencing outcomes, and thus provide a recommendation.

## Materials and methods

We retrospectively reviewed the medical records of patients operated on for DDH between January 2010 and December 2018 in the Department of Paediatric Orthopaedic and Traumatology of the University Hospital Hassan II – Fez (Morocco). The study included patients operated on for late presentation of DDH or after the failure of conservative treatment with a minimum follow-up of 3 years. Patients with incomplete records or loss of follow-up, patients with an associated neuromuscular disorder, syndromic presentation, and operated on for residual dysplasia of the acetabulum after closed reduction were excluded. Among 425 patients operated on for non-traumatic dislocation of the hip, 301 patients (including 113 bilateral, 108 left, and 80 right DDH) met our selection criteria. A total of 414 hips were included in this study. The mean age at the time of surgery was 34.6 months (14–96 months). There were 52 boys and 249 girls. Thirty-seven hips were operated on after failure of conservative treatment, while 377 hips were for late diagnosis. The mean follow-up period was 6.5 years (3–10 years).

### Surgical technique

Our patients are operated on by three senior surgeons. No traction is made before surgery. All patients are operated on in a supine position, under general anesthesia. The anterior approach of “Smith-Peterson” is used. After spotting and protecting the lateral femoral cutaneous nerve, we pass through the interval between the sartorius and tensor of facia lata. The iliac apophysis is then incised and splitted. The straight tendon of the rectus femoris muscle is disinserted from the anterior inferior iliac spine. Iliopsoas tendon is released from its insertion in the lesser trochanter. Percutaneous tenotomy of the adductors is performed when the operator finds that they are retracted (performed in 367 hips). After “T” capsulotomy, the joint is exposed. We cut the ligamentum teres and the transverse acetabular ligament, and we remove any fibrous/fatty tissue in the acetabular fossa. The capsule is released from the ilium (false acetabulum) until the acetabular rim by a periosteal elevator. The redundant capsule is then excised, and capsulorrhaphy is performed after the reduction of the femoral head. Subtrochanteric femoral shorting was performed by the direct lateral approach of the proximal femur for hips classified Tönnis [7] 3 or 4 in children over 36 months (and over 18 months from January 2015). The length of shortening is equal to the overlap obtained after performing femoral osteotomy and reduction of the head in the acetabulum. The amount of shortening was not recorded. Derotation is performed by external derotation until the patella is centered in the coronal plane. Salter pelvic osteotomy (with fixation of the bone graft by two K-wire) or Dega acetabuloplasty are made for children from 3 to 6 years old and children less than 3 years old when reduction of the femoral head is unstable intraoperatively. The choice between these two procedures (Salter or Dega) depends on the preferences of the operator. For children over 6 years old, “Pol Le Coeur” pelvic triple osteotomy is performed. The patients are immobilized with approximately 20° abduction, 20° internal rotation, and 20° flexion spica cast for 10–12 weeks. The description of operations is detailed in Table 1. After plaster removal, clinical and radiographic control is performed at 1 month, 3 months, 6 months, and 1 year and then every 2 years until the age of 15.

**Table 1:** Summary of surgical techniques.

	% (Number)
Technique	
OR	18% (72 hips)
OR + FO	32% (130 hips)
OR + PO	8% (37 hips)
OR + OF + PO	42% (175 hips)
Overall	414 hips
Type of pelvic osteotomy	
Salter	70% (147 hips)
Dega	19% (40 hips)
Triple osteotomy	11% (25 hips)
Overall	212 hips

OR: open reduction; FO: femoral osteotomy; PO: pelvic osteotomy.

From the medical records, we have collected the preoperative clinical and radiographic data including the age at surgery, previous orthopedic treatment and its type, side, Tönnis grade, acetabular index (AI) and surgical data. At the last follow-up, the clinical data were evaluated according to the McKay classification [8]. McKay grade I and II are considered good results. On the radiograph, the hips were classified at the last follow-up according to the Severin classification [9]. Severin grade I and II results are considered acceptable, whereas grades III to VI are not. Redislocation, reduction failure, and residual dysplasia were noted and AVN was assessed according to Kalamchi and MacEwen classification [10]. The acetabular index was also measured at the last follow-up.

### Statistical analysis

Descriptive statistics were used to describe patient and treatment characteristics. Frequencies were used for qualitative variables, while means and standard deviations were used for quantitative variables. The study of the link between the different variables and outcomes was carried out using the chi-squared test or the Fisher test for the qualitative variables, while the analysis for the quantitative variables was performed using Student’s test.

## Results

**Figure 1:**
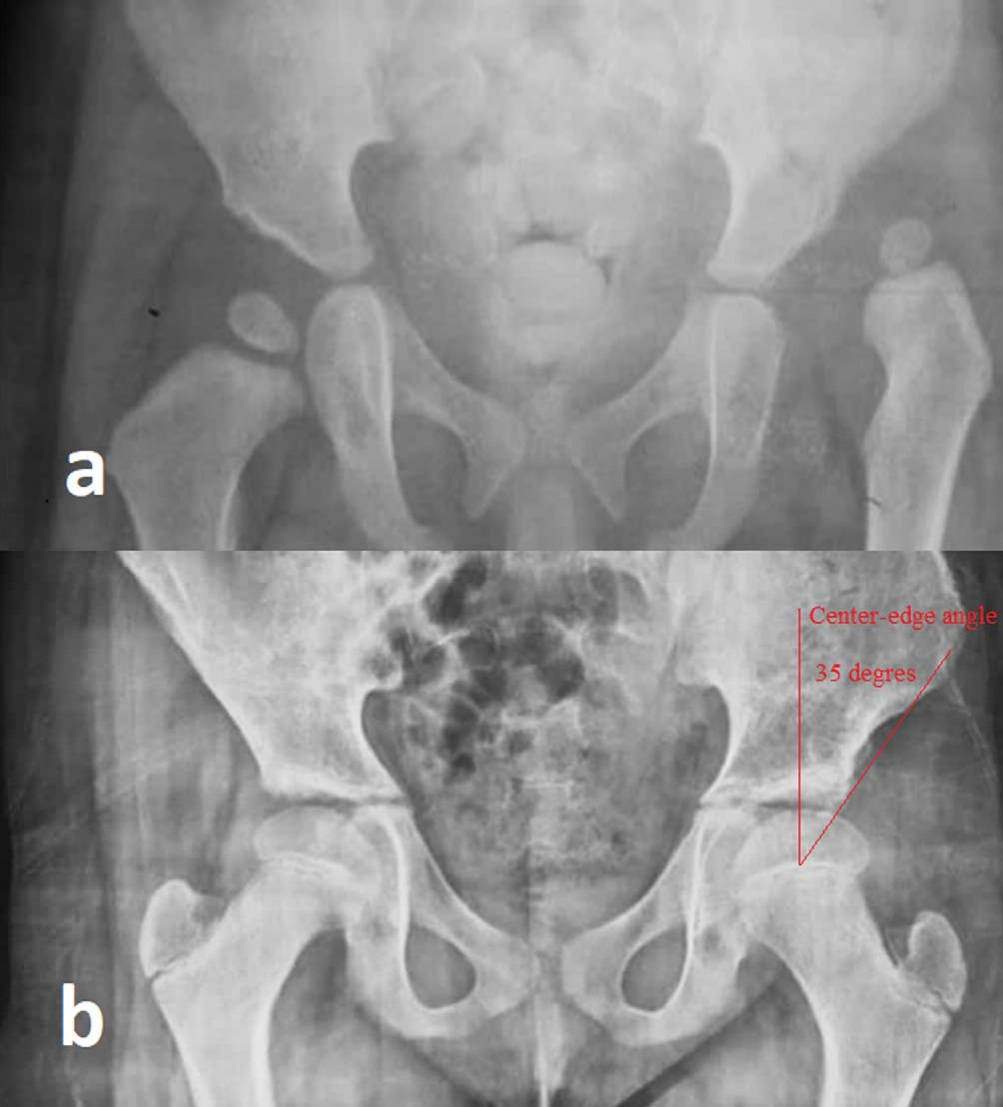
(a) Preoperative radiograph of a girl 1.5 years old of age with right DDH; (b) radiograph at the age of 8 with center-edge angle(CEA) of 35° (Severin score at I) at 6.5 years follow-up after open reduction.

**Figure 2:**
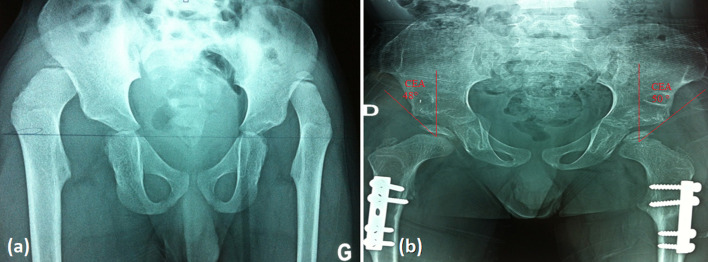
(a) Preoperative radiograph of a boy 5 years old of age with bilateral DDH; (b) radiograph at 4 years follow-up after open reduction combined to femoral osteotomy and Dega pelvic osteotomy (Severin score at I on both sides, CEA at 50° on the right and 48° on the left).

**Figure 3:**
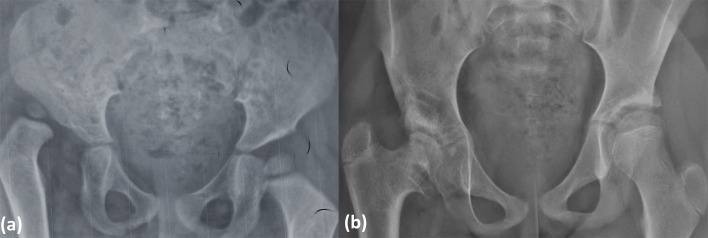
(a) Preoperative radiograph of a girl 2.5 years old of age with right DDH; (b) radiograph at the age of 11 (8.5 years follow-up after open reduction combined to Dega pelvic osteotomy) showing AVN type III of Kalamchi and Macewen with coxa vara.

On 414 hips operated on, 299 (72.2%) were Tönnis 4, 86 (20.8%) Tönnis 3 and 29 (7%) Tönnis 2. The AI was higher than 25° in 382 hips (92%). At the last follow-up, 331 hips (80.2%) had good clinical results (McKay grade I and II), 319 (77%) of satisfactory radiological results corresponding to Severin I or II (Figures 1 and 2). The AI measured on the last follow-up radiograph was ≤25° in 350 hips (84%). The mean AI was 23° (range 18–45°). AVN was noted in 83 hips (20%) distributed as followed: 21 (5.1%) type 1, 22 (5.3%) type 2, 30(7.2%) type 3, and 10 (2.4%) type 4 (Figure 3). Redislocation was noted on 53 hips (12%) of which 42 were reoperated, and residual dysplasia on 24 hips (6%) of which 8 were reoperated. Overall: 293 hips (72%) had stable reduction without AVN with good clinical and radiological outcomes.

Outcomes according to age groups showed that the rate of AVN, redislocation and poor clinical and radiological outcomes increased with age (Table 2), but these results are not statistically significant. Bilaterality (*p* 0.268), previous orthopedic treatment (*p* 0.83) and Tönnis grade (*p* 0.56) are not risk factors for AVN. Results according to the type of surgical treatment were analyzed (Table 3): It was noted that the clinical outcomes are better (*p* 0.019), and the risk of AVN decreases significantly (*p* < 0.0001) when a femoral osteotomy is performed. There were better radiological results (Severin I–II) when pelvic osteotomy was performed (*p* 0.001). Redislocation and residual dysplasia are less frequent in the case of pelvic osteotomy, but this result is not statistically significant. In results according to the type of surgery in the age groups 18–36 months and 36–72 months (Table 4). We note in the first group that there was a statistically significant decrease in AVN risk when femoral shortening osteotomy was performed (*p* < 0.0001) and a decrease of redislocation (*p* 0.061) and residual dysplasia (*p* 0.001) in case of the associated pelvic osteotomy. The radiological results according to Severin classification were better when femoral and pelvic osteotomy was associated (*p* < 0.0001). Clinical outcomes were better when femoral osteotomy was performed (*p* 0.022). In the age group 36–72 months, we note a decrease of AVN (*p* 0.002) and good radiological outcomes (*p* 0.05) when pelvic and femoral osteotomies were associated. Overall good results (stable reduction without AVN or residual dysplasia with good clinical and radiological outcomes) of the two groups were described in Table 4. In age group under 18 and above 72 months, the analysis was not performed since almost patients had the same treatment (OR alone in the first group and OR associated with femoral osteotomy and “Pol-Le-Coeur” pelvic osteotomy in the second).

**Table 2:** Description of results in age group.

Age group (months)	Number	AVN *n* (%)	McKay III–IV *n* (%)	Severin III–IV *n* (%)	Redislocation *n* (%)
≤18	31	1 (3.2%)	2 (6.5%)	2 (6.5%)	1 (3.2%)
18–36	270	54 (20%)	54 (20%)	59 (21.9%)	38 (14.1%)
36–72	90	20 (22.2%)	21 (23.3%)	20 (22.2%)	11 (12.2%)
≥72	23	8 (34.8%)	6 (26.1%)	14 (60.9%)	3 (13%)

**Table 3:** Outcomes versus type of treatment.

Type of treatment	AVN (%)	Severin III–IV (%)	McKay III–IV (%)	Redislocation (%)	Residual dysplasia (%)
OR	30.6%	22.2%	23.6%	15.3%	6.9%
OR + FO	8.5%	34.6%	18.5%	16.2%	13.1%
OR + PO	56.8%	10.8%	33.8%	10.8%	0.00%
OR + OF + PO	16.6%	17.1%	16%	9.7%	1.1%
*P*-value	<0.0001	0.001	0.019	NS	NS

OR: open reduction; FO: femoral osteotomy; PO: pelvic osteotomy; NS: not significant.

**Table 4:** Description of results according to 18–36 and 36–72 months age group and surgical techniques.

Age group (month)	Operation	*n*	AVN *n* (%)	McKay III–IV *n* (%)	Severin III–IV *n* (%)	Redislocation *n* (%)	Residual dysplasia *n* (%)	Overall good results* *n* (%)
18–36	OR	49	11 (22%)	13 (26%)	18 (36%)	10 (20%)	6 (12%)	24 (48%)
	OR + FO	111	10 (9%)	21 (19%)	39 (35%)	21 (19%)	16 (14%)	73 (66%)
	OR + PO	25	6 (24%)	9 (36%)	6 (24%)	5 (20%)	0	16 (64%)
	OR + OF + PO	85	5 (6%)	10 (11%)	11 (13%)	1 (1%)	0	73 (86%)
36–72	OR	3	2 (66%)	2 (66%)	2 (66%)	1 (33%)	0	1 (33%)
	OR + FO	17	5 (29%)	3 (17%)	3 (17%)	1 (6%)	1 (0.5%)	12 (70%)
	OR + PO	7	3 (42%)	4 (57%)	1 (14%)	2 (28%)	1 (14%)	3 (42%)
	OR + OF + PO	63	10 (16%)	12 (19%)	7 (11%)	8 (12%)	0	51 (81%)

* Stable reduction without AVN or residual dysplasia with good clinical and radiological outcomes.

We have analyzed the risk of redislocation and residual dysplasia in the case of hips with acetabular index upper than 25° in which pelvic osteotomy was done or not. The rate of residual dysplasia was significantly higher when pelvic osteotomy was not performed (*p* < 0.0001). Redislocation was also frequent but statistically it remains insignificant (*p* 0.37).

## Discussion

DDH is a common pathology in pediatric orthopedics that should be screened and diagnosed as soon as possible [11]. The incidence of DDH is estimated at 1 per 400 births in UK [12], 6 per 1000 in France [13] and 1–5 per 1000 births in the USA [11]. In 2010, the incidence of DDH diagnosed after the age of 1 year was 8.4 per 100,000 births in France [14]. In our context this rate is higher. We operated on average 52 DDHs per year. The higher number reflects the rate of late diagnosis in our context despite screening efforts. It is also due to the extension of the surgical indication to young children (<18 months) who could be managed by conservative treatment (traction and closed reduction). This therapeutic choice is due to the constraints of this treatment (long hospital stay with reduced bedding capacity and the resulting economic cost).

We report clinical and radiological outcomes of a large series of surgical treatments of DDH with several variables (age, bilaterality, previous orthopedic treatment, and different types of surgery). Nonetheless, our series has some limitations: first, it is a retrospective study and then, the duration of follow-up is relatively short, hence the need to follow-up patients and perform studies to assess long-term results.

In our series, 293 hips (72%) had stable reduction without AVN with good clinical and radiological outcomes. This compares favorably with previous reports in the literature [1, 6, 12, 15] (Table 5).

**Table 5:** Outcomes of other series of literature.

Authors (Country)	Number of cases	Period	Mean age (months)	Mean follow-up (years)	Good clinical results McKay I–II (%)	Good radiological results Severin I–II (%)	AVN (%)	Redislocation (%)
Castañeda et al. (Mexico) [6]	712	2000–2010	25.2	9.3	87%*	80%	14%	2%
Ning et al. (China) [1]	864	2005–2010	69.6	6.2	79.4%	84.7%	27.4%	1.6%
McFarlane et al. (UK) [12]	47	2004–2008	25	7	–	89%	13%	15%
Ahmed et al. (Egypt) [15]	26	2005–2006	14.7	3.9	89%	77%	–	3.8%
Our series (Morocco)	414	2010–2018	34.6	6.5	80.2%	77%	20%	12%

* According to children’s hospital Oakland hip evaluation score (CHOHES).

In clinical outcomes, 80.2% good results were found (McKay score ≤ 2). Many authors [16, 17] report poor clinical results for children operated on at a late age. In our series, no significant difference was found for age groups over 18 months. However, children under 18 months have better clinical results. We did not find a significant difference in clinical outcomes between unilateral and bilateral DDH. Wang et al. [17] compared in their study the results of unilateral and bilateral DDH and found the same results.

Radiologically, 81.2% good outcomes were found. This rate is similar to results found in other series, ranging from 65% to 89% [12, 16, 17]. Hips operated between 18 and 36 months and beyond 6 years, and cases without pelvic osteotomy had poor radiological results. However, in a meta-analysis published in 2016 [18], authors noted that open reduction alone is associated with a high probability of good radiological results (97%), compared to a reduction associated with a pelvic and femoral osteotomy (83%).

The AVN rate found in our series is 20% (83 hips). This risk was greater in children over 3 years old and in children over 18 months who did not have femoral shortening. The risk of AVN has been studied by several authors: Glorion [19] suggests that this necrosis can be iatrogenic. It may be due to excessive traction on the posterior vessel-carrier blade or its direct surgical attack, or following an extreme hip abduction position in immobilization. It can also be due to hypertension on the epiphysis if the femur has not been shortened. Sankar et al. [20] recommend a femoral shortening osteotomy when the height of the head exceeds the width of the acetabulum by more than 30% for children over 18 months. This necrosis can also be due to previous vascular lesions of a dislocated hip following failures of closed reduction. Other studies [12, 21] report that age at surgery, grade of Tönnis, and immobilization cast in an inappropriate position are also risk factors for AVN.

The incidence of redislocation and residual dysplasia reported in the literature is 1–14% [1, 16]. The risk factors reported are multiple: age, bilaterality, femoral anteversion, inappropriate pelvic osteotomy, Tönnis grade, faulty hip position during postoperative immobilization, and errors in the surgical technique [11, 17]. In our series, the rate of redislocation and residual dysplasia was 15%. This risk was correlated with advanced age, Tönnis grade, and non-achievement of pelvic osteotomy in patients with an acetabular index higher than 25°. Regarding the type of pelvic osteotomy, there is no significant difference in the radiological results according to the Severin classification between Salter osteotomy and Dega acetabuloplasty, and the improvement in the acetabular index is similar. The triple osteotomy has only been used for children over 6 years old. The choice between the different pelvic osteotomy techniques remains controversial. In a mid-term radiographic comparison of Dega and Salter osteotomies, López-Carreño et al. [22] found better improvement in the acetabular index after Dega osteotomy, especially in patients under 8 years of age. However, in a study of patients who had a Dega or Salter osteotomy, poor clinical and radiographic results were observed in patients less than 4 years old who had a Dega osteotomy [23]. This finding can be explained by the technical difficulty of this osteotomy in a very young child with a thin iliac bone [4].

## Conclusion

No international consensus regarding surgical treatment of DDH has been established. Our analysis showed a decrease in AVN with femoral shorting and a decrease in the rate of residual dysplasia in the event of a pelvic osteotomy for hips with an acetabular index greater than 25°. We recommend at the end of our results

- A femoral shortening osteotomy for high dislocations (Tönnis 3 or 4) for children over 18 months.
- A pelvic osteotomy for children over 36 months or over 18 months with an acetabular index > 25°.
